# Europium Nanoparticles-Based Fluorescence Immunochromatographic Detection of Three Abused Drugs in Hair

**DOI:** 10.3390/toxics11050417

**Published:** 2023-04-29

**Authors:** Shujuan Xu, Biao Ma, Jiali Li, Wei Su, Tianran Xu, Mingzhou Zhang

**Affiliations:** 1 Zhejiang Provincial Key Laboratory of Biometrology and Inspection & Quarantine, China Jiliang University, Hangzhou 310018, China; xushujuan31@163.com (S.X.); 16a0701109@cjlu.edu.cn (B.M.); 2 Hangzhou Quickgene Sci-Tech. Co., Ltd., Hangzhou 310018, China; qjc1993@126.com; 3 Wenzhou MeiZhong Medical Laboratory, Wenzhou 325000, China; suwei80@sina.com; 4 College of Life Science, China Jiliang University, Hangzhou 310018, China; xiaotianrantongxue@163.com

**Keywords:** europium nanoparticles, fluorescence immunochromatographic assay, triple detection, drug, hair

## Abstract

Drug abuse is becoming increasingly dangerous nowadays. Morphine (MOP), methamphetamine (MET) and ketamine (KET) are the most commonly abused drugs. The abuse of these drugs without supervision can cause serious harm to the human body and also endanger public safety. Developing a rapid and accurate method to screen drug suspects and thus control these drugs is essential to public safety. This paper presents a method for the simultaneous quantitative detection of these three drugs in hair by a europium nanoparticles-based fluorescence immunochromatographic assay (EuNPs−FIA). In our study, the test area of the nitrocellulose membrane was composed of three equally spaced detection lines and a quality control line. The test strip realized the quantitative analysis of the samples by detecting the fluorescence brightness of the europium nanoparticles captured on the test line within 15 min. For the triple test strip, the limits of detection of MOP, KET and MET were 0.219, 0.079 and 0.329 ng/mL, respectively. At the same time, it also showed strong specificity. The strip was stable and could be stored at room temperature for up to one year, and the average recovery rate was 85.98–115.92%. In addition, the EuNPs−FIA was validated by high-performance liquid chromatography (HPLC) analysis, and a satisfactory consistency was obtained. Compared to the current immunochromatographic methods used for detecting abused drugs in hair, this method not only increased the number of detection targets, but also ensured sensitivity, improving detection efficiency to a certain extent. The approach can also be used as an alternative to chromatography. It provides a rapid and accurate screening method for the detection of abused drugs in hair and has great application prospects in regard to public safety.

## 1. Introduction

The incidence of drug overdose deaths has increased continuously and quickly over the past 40 years, affecting people all over the world [1]. The phenomenon of narcotic drug and psychotropic substance abuse has emerged as one of the most serious threats to human health in the times of calmness. The United Nations Office on Drugs and Crime (UNODC) has published the World Drug Report 2022 [2]. According to the report, in 2020, an estimated 284 million people worldwide aged 15–64 years had used a drug within the last 12 months, representing a 26 percent increase from 2010. The global drug epidemic situation will worsen as the epidemic spreads; at the same time, with the progress of society, more and more new drugs have emerged. Drug testing has also become the focus of research.

According to the data of the Academy of Forensic Sciences (Shanghai, China) from February 2017 to December 2017 released by Pan [3], of the 6787 drug abuse analysis cases, amphetamines were the most frequently abused drugs, followed by opiates, ketamine and cocaine. Opium and opioids (primarily morphine) are the world’s most abused illicit drugs. They belong to the class of strong analgesics, and their analgesic effect is both efficient and selective. They are highly addictive and impossible to break free from for the rest of one’s life [4]. Methamphetamine, also known as “ice”, is a new synthetic drug that has become the second most abused illicit drug. It causes severe mental disorders and organ damage, particularly to the central nervous system and cardiovascular system [5,6]. Ketamine was discovered in the 1960s, then made available to the public in 1970 [7]. It is extensively utilized during surgical procedures in humans and animals for its anesthetic effect [8]. Ketamine has been found to play an essential role in the treatment and adjuvant therapy of depressive disorders in recent years [9]. Due to its strong hallucinogenic effect and certain psychological dependence, repeated consumption leads to serious addiction [10]. In addition, the simultaneous use of multiple drugs is common among drug users [11]. The large-scale use of these drugs without supervision may cause serious public safety problems, and even death. To enable on-site drug detection in clinical or forensic contexts, highly sensitive and accurate technologies which are low-cost, portable and reusable are urgently needed.

**Figure 1:**
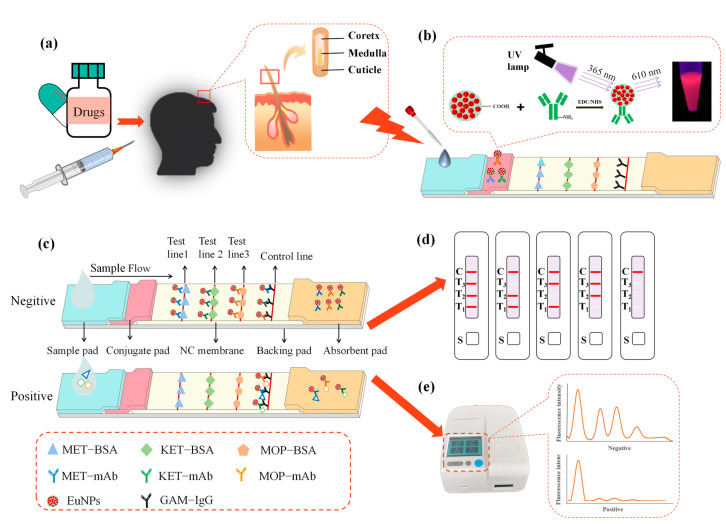
The EuNPs−FIA detection principle. (**a**) Hair structure. (**b**) Appearance of the strip under 365 nm irradiation. (**c**) The EuNPs−FIA detection schematic. (**d**) Schematic diagram of the EuNPs−FIA results. (**e**) Test results of the EuNPs−FIA reader. (MET−BSA: methamphetamine-bovine serum albumin; KET−BSA: ketamine-bovine serum albumin; MOP−BSA: morphine-bovine serum albumin; MET−mAb: methamphetamine monoclonal antibody; KET−mAb: ketamine monoclonal antibody; MOP−mAb: morphine monoclonal antibody; EuNPs: europium nanoparticles; GAM−IgG: goat anti-mouse IgG).

The main samples used to detect abused drugs include blood [12], saliva [13], urine [14] and hair. However, the majority of medications are quickly metabolized in the blood or urine and are only detectable after a few hours, up to a few days. Nevertheless, the same substances are fixed in the structure of hair, leaving a history of consumption that can be found in a matter of weeks or months [15]. Hair became popular as an analytical sample for drug detection in 1979, when a radioimmunoassay established the existence of opioid chemical compounds in human hair [16]. Hair is a filiform structure composed of a cuticle, cortex and medulla (Figure 1a). Drugs and their metabolites can enter the hair shaft during its development [17]. Without additional preventive measures, it is easy to store and transport hair samples, and it is difficult to adulterate samples. At the same time, collecting hair samples is less invasive than collecting blood or urine samples. For these reasons, hair is an important tool in forensic and criminal investigations [18].

Gas chromatography/mass spectrometry (GC/MS) [19], liquid chromatography–tandem mass spectrometry (LC−MS/MS) [20], high-performance liquid chromatography (HPLC) [21], UPLC−MS/MS [22], nano−HPLC coupled with tandem mass spectrometry (nano−HPLC−Chip−MS/MS) [23], surface plasmon resonance (SPR) [24], surface-enhanced raman scattering (SERS) [25,26] and electrochemical methods are commonly used to detect drugs. These technologies have a high accuracy, but require a large number of fluid samples, expensive equipment and high skill. Immunoassays have attracted extensive attention in drug detection in recent years due to their simple operation, high efficiency, high sensitivity, strong specificity and applicability to large-scale detection. Examples include enzyme-linked immunosorbent assay (ELISA) [27], capillary electrophoresis (CE) immunoassay [28], chemiluminescence immunoassay [29] and lateral flow immunoassay (LFIA) [30,31]. LFIA is a method that combines immunoreactivity with chromatography, the most common form of which is based on gold nanoparticles [32]. However, the poor repeatability and low sensitivity of colloidal gold nanoparticles also limit its applications. In order to meet the requirements of a more accurate and sensitive analysis, the combination of fluorescent microspheres as labelling probes and portable strip readers has become a research hotspot in recent years. Quantum dots (QDs), carbon nanodots-silica [33], upconverting nanoparticles [34], fluorescein isothiocyanate (FITC) [35] and lanthanide-labeled fluorescent nanoparticles [36], as substitutes for colloidal gold, have been widely used in applications in clinical detection [37], molecular biology [38] and other related fields. Europium nanoparticle (EuNP) luminous material has been widely employed because of its benefits of ultra-long emission half-life, massive Stokes shift, longer measurement duration, removal of background fluorescence and better detection sensitivity [39]. A few studies have reported using fluorescent nanoparticles to simultaneously detect multiple abused drugs [40,41,42].

In modern clinical diagnosis practice, the demand for the rapid quantitative or qualitative detection of various substances in samples is increasing. In this paper, we propose a triple fluorescence immunochromatographic method based on europium nanoparticles for the simultaneous quantitative detection of MOP, MET and KET. By detecting multiple abused drugs in a triple test strip, this screening method provides a shorter analysis time and higher efficiency, and can be used as an alternative to chromatography. In the future, the rapid detection of multiple targets in one sample has great application prospects.

## 2. Materials and Methods

### 2.1. Materials

MOP, KET, MET, cannabidiol, phenobarbital, diazepam, mephedrone, caffeine, taurine, papaverine, (1R, 2S)-(-)-ephedrine hydrochloride, pseudoephedrine hydrochloride, tetrahydrocannabinol, norketamine, sodium pentachlorophenol, R(+)-cathinone hydrochloride, methcathinone, tetrahydro-cannabinolic acid, methadone, methoxyphenamine, phenylpropanolamine hydrochloride, methylenedioxymethamphetamine, 6-monoacetylmorphine and F-ketamine standards were obtained from the Physical Evidence Center of the Ministry of Public Security (Beijing, China). Morphine bovine serum albumin (MOP−BSA), methamphetamine bovine serum albumin (MET−BSA), ketamine bovine serum albumin (KET−BSA), morphine monoclonal antibody (MOP−mAb), methamphetamine monoclonal antibody (MET−mAb) and ketamine monoclonal antibody (KET−mAb) were purchased from Hangzhou Quickgene Biotech Co., Ltd. (Hangzhou, China). The 2-morpholinoethanesulphonic acid (MES), 1-(3-Di-methylaminopropyl)-3-ethylcarbodiimide hydrochloride (EDC), N-hydroxysuccinimide (NHS), goat anti-mouse IgG (GAM−IgG) and Tween−20 were purchased from Sigma-Aldrich (St. Louis, MO, USA). Carboxylate-modified EuNPs (200 nm diameter) were purchased from Suzhou VDO Biotech Co., Ltd. (Suzhou, China). Nitrocellulose (NC) membranes, sample pads, conjugate pads, backing card and absorbent pads were purchased from Zhejiang Dian Biotechnology Co., Ltd. (Hangzhou, China). Phosphate-buffered saline (PBS, 0.01 M, pH 7.4), borate buffer solution (BBS, 0.05 M, pH 8.0) and other chemicals were obtained from Sinopharm Chemical Reagent Co., Ltd. (Shanghai, China).

Positive hair samples were collected from people who had a history of drug abuse in the past three months and were provided by Wenzhou MeiZhong Medical Laboratory (Wenzhou, China). Practitioners used gas chromatography (GC) (GA/T 1008.2−2013 [43]; GB/T 29637−2013 [44] and GB/T 29636−2013 [45]) to verify that these samples were positive and provided relevant information, including sex, age and test results (Supplementary Materials, Table S1). The test results were judged according to the Ministry of Public Security of the People’s Republic of China’s specification for hair sample testing of drug-abuse-related personnel.

The blank samples were collected from our volunteers, and these negative samples were tested using the GC method. The detection results were consistent with the previous interpretation method. According to the recommendations for drug detection in hair by the Society of Hair Testing (SOHT) [46], the sample should be cut with scissors from the posterior vertex region of the head, as close as possible to the scalp. The proximal portion of hair (approximately 3 cm) was used for analysis, since this region has the least variation in growth rate. All samples were stored at room temperature.

### 2.2. Instruments

We used a XYZ3000 distribution platform and CM2000 guillotine cutter to prepare test strips (BioDot, Irvine, CA, USA). A Hitachi F−4500 fluorescence spectrometer system (Hitachi, Tokyo, Japan) was used to record the fluorescence spectra. An FIC−S2011−B14 Dry Fluorescence Immunoassay Analyzer was used to read the test strips (Suzhou Hellman Precision Instruments, Suzhou, China). The immunochromatographic strip results were compared with the data obtained using an Agilent 1100 high-performance liquid chromatography system (Agilent Tech, Santa Clara, CA, USA). Hair was crushed with a ball mill (Beijing Wanfu Intelligent Technology Co., Ltd., Beijing, China).

### 2.3. Preparation of Fluorescent Probes

According to the specific scheme of europium nano-labeled antibodies in our laboratory in the early stages and the related literature reports [47], the specific operation was as follows: 2 mg of fluorescent microspheres was added to 1 mL of MES (0.05 M, pH 5.0) solution and well mixed, centrifuged for 20 min at 12,000 rpm/min; then, the supernatant was removed and we added 1 mL MES, mixed and sonicated. We repeated the appeal operation and washed EuNPs three times. After that, we added 200 μL of MES solution, stirred thoroughly and sonicated. We used the MES solution to produce 10 mg/mL NHS, and 15 μL was added to the sonicated fluorescent microspheres. We prepared 15 mg/mL of EDC with ultrapure water; then, 2 μL of 15 mg/mL of EDC was added to the previously mentioned fluorescent microspheres. In order to enhance antibody attachment, the carboxyl group on the EuNP surface was incubated for 30 min with moderate shaking. Excessed EDC was removed after activation by centrifugation at 12,000 rpm/min for 25 min. We added 1 mL PBS solution (0.01M, pH 7.4) to the precipitate, mixed well and then added MOP−mAb. After that, the fluorescent microspheres were activated and mixed for 2 h. An amount of 50 μL of BB (0.05M, pH 8.0 containing 10% (*w*/*v*) BSA) was used to inhibit the remaining active sites of the complex. Finally, fluorescent compounds were dissolved in 50 μL of BB (0.05 M, pH 8.0, including 1% (*w*/*v*) BSA and 0.5% (*v*/*v*) Tween−20) and kept at 4 °C in the dark for further use. EuNPs−KET−mAb and EuNPs−MET−mAb were prepared using a similar method.

### 2.4. Preparation and Assembly of the Single-Channel Strip

The immunoassay strip was made up of five pieces, as illustrated in Figure 1: an absorbent pad, a backing pad, a conjugate pad, a sample pad and a nitrocellulose membrane (NC).

The conjugate pad and the sample pad were both composed of the same gauge fiberglass. The sample pads were cut into 30 cm long and 2 cm wide strips before being preprocessed with 5 mL of PBS (0.05 M, pH 7.4, containing 2% (*w*/*v*) sucrose, 0.5% (*w*/*v*) BSA and 0.5% (*v*/*v*) Tween−20). The artificial antigens (MOP−BSA, KET−BSA or MET−BSA) and goat anti-mouse IgG (1 μL/cm) were sprayed on the NC membrane through the XYZ3000 distribution platform to become the test line (T line) and the control line (C line). Then, we dried the NC films for 2 h in an oven at 37 °C. In the BBS buffer (0.05 M, pH 8.0, including 8% (*w*/*v*) sucrose and 1% (*w*/*v*) BSA), europium nanoparticles were conjugated with monoclonal antibody (EuNPs−MOP−mAb, EuNPs−KET−mAb or EuNPs−MET−mAb). They were sprayed on the conjugate pad and placed in an oven at 37 °C to dry for 24 h. The whole immunochromatography system was built after the aforementioned components were processed. We assembled the sample pad, conjugate pad, NC membrane and absorption pad in the sequence specified, leaving a 2 mm space between each component, and then we overlapped and sliced them to a width of 3 mm. Then, the test strips were put into a cartridge for further investigation. Cutting and packing were conducted in a separate room with a temperature of 20–22 °C and a relative humidity of less than 30%.

### 2.5. Test Strip Detection Process

A total of 80 μL of standard solution or sample solution was dropped on the sample pad for EuNPs−FIA. Then, the findings were viewed under a portable 365 nm UV light within the immune response time. Simultaneously, we inserted the test strip into the fluorescent dry immunoassay analyzer to read the data and record the fluorescence intensity on the T and C lines.

### 2.6. Preparation of the Triple-Immunochromatographic Test Strip

These were prepared using the single-component immunoassay strip preparation method. We increased the number of T lines on the NC membrane based on the single-channel immunological test strip. We sprayed MET−BSA, KET−BSA, MOP−BSA and goat anti-mouse IgG on the NC membrane (Figure 1b,c). We diluted EuNPs−MET−mAb, EuNPs−KET−mAb and EuNPs−MOP−mAb with the BBS solution. After that, we sprayed them on the conjugate pad according to a certain concentration.

### 2.7. Optimization of Parameters of the Triple-Immunochromatographic System

Placing multiple analytes on a strip may affect the color rendering results. In order to select the best location of the binding area, the color rendering intensity of MOP, MET and KET were tested in three different binding areas on the basis of the single optimization results (referred to in Supplementary Materials, Figure S1). According to the obtained data, we selected the best combination position of the three analytes.

### 2.8. Performance Evaluation of Triple-Immunochromatographic Strips

#### 2.8.1. Sensitivity and Specificity

To create varying concentrations of the mixed standard solutions, the three standards (MOP, KET and MET) were diluted with the BBS buffer (0.01 M, pH 8.0). The amounts of MOP/KET/MET were 0.2/0.2/0.2 ng/mL, 0.4/0.4/0.4 ng/mL, 0.6/0.6/0.6 ng/mL, 0.8/0.8/0.8 ng/mL, 1/1/1 ng/mL, 2/2/2 ng/mL, 4/4/8 ng/mL, 10/10/10 ng/mL, 12/12/20 ng/mL and 14/14/25 ng/mL. Under optimal conditions, MOP, KET and MET were measured simultaneously using triple-immunochromatographic strips, and the fluorescence values of the T or C lines were read by a fluorometric reader. A standard curve was created, and a linear equation was fitted. The half-maximal inhibitory concentration (IC_50_) and the limit of detection (LOD) were utilized as quantitative measures to evaluate the performance of the test strip. The linear range of this analysis was set at the concentration that results in 10–80% inhibition.

The specificity of the triple-test strip was determined by testing a negative sample or a standard (4 ng/mL) containing only one or more abused drugs, and observing whether the three test lines produced a crossover result. In order to further evaluate the specificity of the strip, other drugs were tested, including cannabidiol, phenobarbital, diazepam, mephedrone, caffeine, taurine, papaverine, (1R, 2S)-(-)-ephedrine hydrochloride, pseudoephedrine hydrochloride, tetrahydrocannabinol, norketamine, sodium pentachlorophenol, R(+)-cathinone hydrochloride, methcathinone, tetrahydro-cannabinolic acid, methadone, methoxyphenamine, phenylpropanolamine hydrochloride, methylenedioxymethamphetamine, 6-monoacetylmorphine and F-ketamine. For cross-reactivity investigation, we diluted each medication to 500 ng/mL and recorded the fluorescence intensity using the reader.

#### 2.8.2. Stability

According to the stability inspection method of in vitro diagnostic test strips, we put the test strips in an oven at 50 °C for 21 days for accelerated destruction stability test to detect the stability of the reagent card.

#### 2.8.3. Precision and Accuracy

To evaluate the precision and accuracy of the triple-immunochromatographic test strip, eight groups from the same batch were selected for negative detection by using a manner similar to that of the single strips. The inter-batch experiment was conducted with eight strips from different batches. We recorded the fluorescence intensity and then determined the inter-assay and intra-assay coefficient of variation. The process was repeated three times.

#### 2.8.4. Spiked Recovery Tests and Actual Sample Analysis

The spiked samples were analyzed by HPLC and EuNPs−FIA to confirm the reliability. To create spiked samples, negative hair samples were spiked with varied amounts of MOP (0.5, 1, 2 and 4 ng/mL), MET (0.5, 1, 2 and 4 ng/mL) and KET (0.5, 1, 2 and 4 ng/mL) standards.

To investigate the practical usefulness of this EuNPs−FIA further, 30 positive samples were analyzed utilizing a multitarget fluorescent immunochromatographic assay, with references to various procedures for test material processing as follows [48]: the hair was firstly washed twice with 10 mL of deionized water, then twice with 5 mL of acetone for 2 min. After drying at room temperature, we cut the hair into small pieces of around 2–3 mm in size, accurately weighed 20 mg of hair and placed it in a dedicated grinding tube. The samples were shaken in 1 mL of methanol in the grinding tube. We used a ball mill to grind the sample for 3 min at a frequency of 3000 r/min, then ultrasound assisted the shaking for 2 h. Then, we transferred 500 μL of the grinding liquid to a centrifuge tube, centrifuged it at 4000 r/min for 5 min, transferred the supernatant to a 0.22 μm organic membrane filtration and finally analyzed the filtrate. We used the above hair treatment method to treat 530 negative hair samples from volunteers, and then used the EuNPs−FIA for detection. All actual samples were analyzed and compared using an Agilent 1100 HPLC system (Agilent Tech, Santa Clara, CA, USA). Phenomenex Kinetex Bighenyl (100 mm 2.1 mm, 1.7 μm) chromatographic column, 0.1% formic acid (1 mmol/L ammonium formate) (phase A) and acetonitrile (phase B) were employed as mobile phases, and the flow rate was 0.1 mL/min. The injection volume was 1 μL, and the column temperature was 35 °C.

### 2.9. Data Analysis

Standard curves, sample means and standard deviations (SD) were determined using OriginPro 9.1 (OriginLab, Northampton, MA, USA) and Microsoft Excel (Microsoft Corporation, Redmond, WA, USA). Schematic diagrams of the test strips were drawn using the Photoshop software (Adobe Systems, San Jose, CA, USA).

## 3. Results and Discussion

### 3.1. Detection Principle of the Immunochromatographic Detection System

This test strip employs the competitive immunochromatography experimental principle. Three distinct antigens are encapsulated on the NC membrane and serve as capture reagents. To serve as detection probes, antibody pairs labeled with EuNPs are sprayed over the conjugate pad. A drop of 80 μL of the test material is placed on the sample pad, and it flows to the other side by capillary action. The fluorescence signal values at the characteristic excitation (365 nm) and emission (610 nm) wavelengths are measured with an immunofluorescence detector. When a negative sample is identified, the conjugated pad’s nano-labeled antibody attaches to the appropriate solid-phase antigen on the nitrocellulose membrane, generating a fluorescent detection line visible under UV light, as illustrated in Figure 1d,e. When the target analyte is present in the sample, it binds to the binding pad’s nano-fluorescent-labeled monoclonal antibody. Excess antibody conjugate continues to travel along the liquid chromatography column due to capillary action and binds to the antigen on the T line, resulting in less fluorescently labeled antibodies on the detection line and a drop in fluorescence intensity. If there are enough target analytes, they will completely prohibit the detection reagent from binding to their respective captures, resulting in no visible detection line at the relevant point on the T line for positive samples. The target analyte content in the test sample is inversely linked with the T line to C line ratio. The lower the target analyte content, the higher the T/C.

### 3.2. Optimization of the Triple-Immunochromatographic System

The single-component test strip was successfully prepared, laying a good experimental foundation for further increasing the number of detection lines at the later stage. The Supplementary Materials provide the optimization details of the single-channel test strip.

To optimize the antigen concentration in the early stage, we conducted the experimental analysis and optimization of the antigen location of the triple fluorescent immunochromatography system; the results are shown in Table 1. When MOP−BSA was in the T_3_ binding region, KET was in the T_2_ binding region and MET was in the T_1_ position, the best color effect was achieved.

### 3.3. Triple-Immunochromatographic Test Strip Performance Evaluation

#### 3.3.1. Sensitivity and Specificity

**Figure 2:**
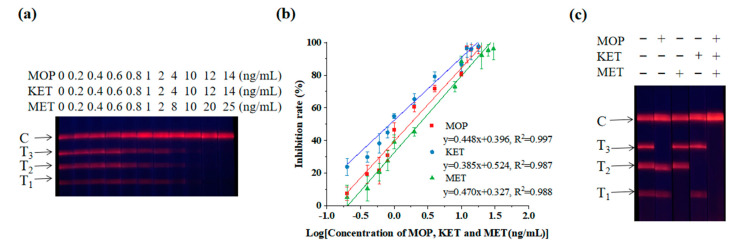
Triple detection of EuNPs−FIA. (**a**) Sensitivity experimental diagram of the triple-test strip. (**b**) Sensitivity curve of the triple-test strip. (**c**) Specific analysis of the triple-test strip. (T_1_: MET−BSA; T_2_: KET−BSA; T_3_: MOP−BSA).

In Figure 2a, it can be observed that as the concentration of the standard increased, the fluorescence intensities of T_1_, T_2_ and T_3_ lines gradually weakened, until they disappeared. The linear equation to detect MOP was y = 0.448x + 0.396, R^2^ = 0.997, LOD = 0.219 ng/mL and IC_50_ = 1.709 ng/mL. The linear equation for detecting KET was: y = 0.385x + 0.524, R^2^ = 0.987, LOD = 0.079 ng/mL and IC_50_ = 0.868 ng/mL. The linear equation for detecting MET was: y = 0.470x + 0.327, R^2^ = 0.988, LOD = 0.329 ng/mL and IC_50_ = 2.341 ng/mL. The results showed a good linear relationship between the logarithm of the drug concentration and the corresponding T/C value (Figure 2b).

The triple test strip was used to detect a standard sample containing only one or more MOP, KET or MET medicines. It showed that there was no cross reactivity between the three substances of abuse (Figure 2c).

**Figure 3:**
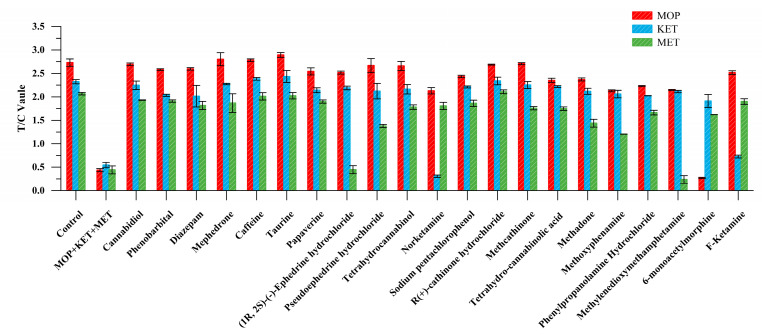
Triple-immunochromatographic strip specificity analysis of other drugs.

Among other drugs, 6-monoacetylmorphine showed cross reaction in the MOP binding region. Norketamine and F-ketamine combined in the KET detection area. Methyenedioxymethamphetamine and (1R, 2S)-(-)-ephedrine hydrochloride showed weak fluorescence signals in the MET detection area. These were mainly due to the structural similarity of these substances (Table S7), but EuNPs−FIA did not react with other drugs, showing great specificity (Figure 3).

#### 3.3.2. Stability

**Figure 4:**
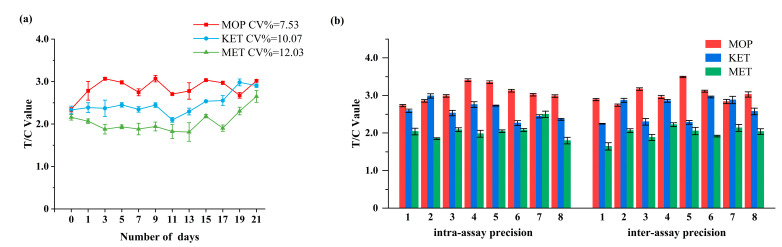
Stability and precision results of the triple-immunochromatographic test strips. (**a**) Triple-immunochromatographic strip accelerated 21 days after the test results at 50 °C. (**b**) Test results of intra-assay and inter-assay experiments of triple immunochromatography. (Intra-assay precision 1–8 represents the same batch of eight different test strips; inter-assay precision 1–8 represents eight different batches of test strips).

Figure 4a depicts the stability test findings of the EuNPs−FIA. The triple-test strip was held at 50 °C for 21 days, and the T/C value almost did not change. The coefficient of variation (CV) was less than 12.03%. It demonstrated that the newly created strip was stable and may be stored at room temperature for up to one year [49].

#### 3.3.3. Precision and Accuracy

The precision of the triple immunochromatographic strip was evaluated by detecting the intra-assay and inter-assay. As shown in Figure 4b, the CV% of the triple-detection strip was less than 10.96%. The small CV value indicated a high accuracy, which meant that the prepared triple strip can meet the requirements of quantitative detection.

#### 3.3.4. Spiked Recovery Tests and Actual Sample Analysis

**Figure 5:**
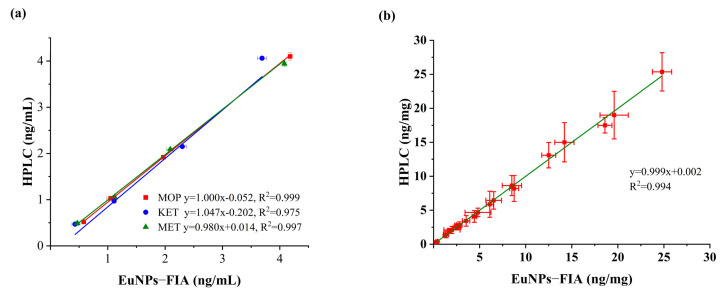
Comparison of EuNPs−FIA and HPLC. (**a**) Comparison of assay recovery for EuNPs−FIA and HPLC. (**b**) Actual sample test comparison for EuNPs−FIA and HPLC.

The accuracy and repeatability of the developed triple immunoassay was checked by testing spiked blank samples. The experiment was repeated six times for each concentration. The results showed that the average recoveries of MOP ranged from 98.61% to 115.92%, with a relative standard deviation (RSD) of less than 6.85% at the spiked concentrations of 0.5–4 ng/mL (Table 2). The average recovery rate of KET was 85.98–111.03%, RSD < 8.19%. The average recovery rate of MET was 94.16–112.34%, RSD < 6.04%. The determination results were verified by HPLC analysis, with a correlation coefficient of R^2^ > 0.97 (Figure 5a).

#### 3.3.5. Actual Sample Detection

In order to further verify the reliability of the EuNPs−FIA quantitative method, 30 actual samples were simultaneously analyzed with EuNPs−FIA and HPLC. To ensure accuracy, each sample was tested three times (Table 3). The results of MOP, KET and MET in the actual samples are highly correlated (Figure 5b). Therefore, the method can be reliably used to detect these three abused drugs in hair.

**Figure 6:**
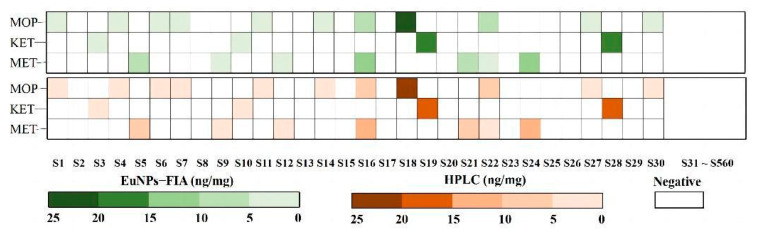
Comparison of the consistency of 560 actual samples detected by EuNPs−FIA and HPLC. (S1 represents sample 1, S2 represents sample 2, and so on).

According to the recommendations for hair testing in forensic cases developed by the International Society for Hair Testing, when the level of morphine, methamphetamine or ketamine is greater than 0.2 ng/mg, a positive result must be produced [46]. The results showed that the negative coincidence rate of 530 samples from volunteers was 100% (Table S6). This result showed that this multiplex test strip analysis was useful for detecting the abused drugs in hair. Compared with the results of 560 actual samples from HPLC analysis, the consistency of the two methods was 100% (Figure 6).

## 4. Conclusions

Nowadays, drug users use more than one drug, with the intention of enhancing or potentiating the drug effects. In this study, a triple fluorescent immunochromatographic strip was successfully developed and optimized for the simultaneous detection of three abused drugs (MOP, KET and MET) in hair samples. The test results can be obtained within 15 min of testing. Through the sensitivity standard curve established by the fluorescence intensity, the results showed that the LOD of MOP, KET and MET was 0.219, 0.079 and 0.329 ng/mL, respectively. In terms of reliability, the EuNPs−FIA showed good accuracy and good consistency with HPLC through the recovery and actual sample detection experiments. Compared with the current immunochromatographic methods for detecting abused drugs, this method effectively improved the issues of a single-target substance and low sensitivity of traditional labelling materials. As an alternative method of chromatography, this screening method can provide help for the detection of clinically abused drugs. In summary, the development of multiple quantitative test strip assays will become a powerful tool for monitoring multiple drugs and use in other health, food safety and environmental tests.

## Figures and Tables

**Table 1:** Antigen location optimization results.

Antigen	T Line Position	Antigen Concentration (mg/mL)	Fluorescence Intensity (CPS ^a^)	T/C Value
MOP−BSA	T_1_	1.5	18,970 ± 0.12	2.05
	T_2_	1.5	14,810 ± 0.56	1.93
	T_3_	1.5	21,176 ± 0.43	2.61
MET−BSA	T_1_	1.0	17,951 ± 0.88	2.35
	T_2_	1.0	15,462 ± 0.22	1.86
	T_3_	1.0	14,117 ± 0.27	1.29
KET−BSA	T_1_	1.5	15,242 ± 0.32	1.62
	T_2_	1.5	18,238 ± 0.78	2.48
	T_3_	1.5	12,131 ± 0.35	2.33

^a^: CPS: counts per second.

**Table 2:** Recovery rate determination of MOP, KET and MET in hair samples by EuNPs−FIA and HPLC analyses.

Standard	Spiked Level (ng/mL)	EuNPs−FIA			HPLC		
		Detected (ng/mL)	Recovery Rate	RSD ^a^ (n = 6)	Detected (ng/mL)	Recover Rate	RSD (n = 6)
MOP	0.5	0.58	115.92%	2.86%	0.52	104.19%	2.03%
	1	1.05	104.87%	6.85%	1.03	103.00%	4.01%
	2	1.97	98.61%	3.23%	1.92	96.00%	2.56%
	4	4.18	104.45%	3.17%	4.10	102.41%	8.66%
KET	0.5	0.43	85.98%	5.45%	0.47	94.69%	3.30%
	1	1.11	111.03%	3.47%	0.97	97.19%	2.17%
	2	2.30	114.83%	7.14%	2.15	107.70%	1.04%
	4	3.69	92.35%	8.19%	4.06	101.41%	3.69%
MET	0.5	0.47	94.16%	4.67%	0.49	98.03%	4.12%
	1	1.12	112.34%	1.81%	1.06	105.66%	3.03%
	2	2.09	104.50%	6.04%	2.09	104.57%	2.01%
	4	4.08	101.88%	3.19%	3.94	98.41%	6.04%

^a^: RSD: relative standard deviation.

**Table 3:** Determination of MOP, KET and MET in hair samples by EuNPs−FIA with HPLC analyses.

	EuNPs−FIA ^a^ (ng/mg)			HPLC ^b^ (ng/mg)		
	MOP	KET	MET	MOP	KET	MET
Hair 1	3.53 ± 0.78	/ ^c^	/	3.43 ± 0.41	/	/
Hair 2	/	/	/	/	/	/
Hair 3	/	1.35 ± 0.49	/	/	1.44 ± 0.29	
Hair 4	0.37 ± 0.05	/	/	0.35 ± 0.14	/	/
Hair 5	/	/	8.75 ± 1.9	/	/	8.20 ± 0.49
Hair 6	1.69 ± 0.32	/	/	2.0 ± 0.08	/	/
Hair 7	2.78 ± 0.47	/	/	2.81 ± 0.31	/	/
Hair 8	/	/	/	/	/	/
Hair 9	/	/	1.22 ± 0.13	/	/	1.30 ± 0.06
Hair 10	/	0.28 ± 0.04	/	/	0.30 ± 0.03	/
Hair 11	2.56 ± 0.22	/	/	2.77 ± 0.28	/	/
Hair 12	/	/	1.99 ± 0.52	/	/	2.10 ± 0.08
Hair 13	/	/	/	/	/	/
Hair 14	0.42 ± 0.04	/	/	0.40 ± 0.01	/	/
Hair 15	/	/	/	/	/	/
Hair 16	6.10 ± 1.92	/	12.5 ± 1.87	5.88 ± 0.38	/	13.10 ± 0.76
Hair 17	/	/	/	/	/	/
Hair 18	24.8 ± 2.82	/		25.36 ± 1.05	/	
Hair 19		18.6 ± 1.13			17.50 ± 0.76	
Hair 20	/	/	/	/	/	/
Hair 21	/	/	6.53 ± 1.28	/	/	6.45 ± 0.85
Hair 22	8.50 ± 1.47		2.54 ± 0.37	8.63 ± 1.05		2.50 ± 0.42
Hair 23	/	/	/	/	/	/
Hair 24	/	/	14.2 ± 2.89	/	/	15.05 ± 1.04
Hair 25	/	/	/	/	/	/
Hair 26	/	/	/	/	/	/
Hair 27	4.43 ± 0.86	/	/	4.09 ± 0.25	/	/
Hair 28	/	19.6 ± 3.05	/	/	19.00 ± 1.53	/
Hair 29	/	/	/	/	/	/
Hair 30	4.82 ± 1.41	/	/	4.68 ± 0.63	/	/

a: EuNPs−FIA repeat assay (n = 3); b: HPLC repeat assay (n = 3); c: not detected.

## Data Availability

Data are contained within the article.

## Supplementary Materials

The following supporting information can be downloaded at: https://www.mdpi.com/article/10.3390/toxics11050417/s1, Figure S1: Identification diagram of the fluorescent probe transmission electron microscope. Figure S2: Optimization of EuNPs and antibody conjugation amount, EuNPs−mAb probe concentration and coating concentration. Figure S3: Optimized reaction time and buffer pH. Figure S4: EuNPs−FIA single-component detection. Table S1: Information on 30 positive samples. Table S2: Comparison of the published rapid detection methods for abused drugs. Table S3: Precision test results of single−MOP immunochromatographic test strips. Table S4: Precision test results of single−KET immunochromatographic test strips. Table S5: Precision test results of single−MET immunochromatographic test strips. Table S6: Testing results of 530 negative hair samples. Table S7: Molecular structure of abused drugs.
