# Supplementary material for: Europium Nanoparticles-Based Fluorescence Immunochromatographic Detection of Three Abused Drugs in Hair
Source: Toxics. 2023 Apr 29;11(5):417. doi: 10.3390/toxics11050417 (PMC10224117; doi:10.3390/toxics11050417)
Supplement: Supplementary file 1 [file toxics-11-00417-s001.zip › toxics-2323385-supplementary.pdf]

Supplementary Material

Europium Nanoparticles-Based Fluorescence Immunochromatographic Detection of Three Abused Drugs in Hair

Shujuan Xu [1,†], Biao Ma [1,†], Jiali Li [2], Wei Su [3], Tianran Xu [4] and Mingzhou Zhang [1,*]

[1] Zhejiang Provincial Key Laboratory of Biometrology and Inspection & Quarantine, China Jiliang University, Hangzhou 310018, China; xushujuan31@163.com (S.X.); 16a0701109@cjlu.edu.cn (B.M.)
[2] Hangzhou Quickgene Sci-Tech. Co., Ltd., Hangzhou 310018, China; qjc1993@126.com
[3] Wenzhou MeiZhong Medical Laboratory, Wenzhou 325000, China; suwei80@sina.com
[4] College of Life Science, China Jiliang University, Hangzhou 310018, China; xiaotianran-tongxue@163.com
* Correspondence: zmzcjlu@cjlu.edu.cn; Tel.: +86-571-86914476; Fax: +86-571-86914510
† These authors contributed equally to this work.

Wenzhou MeiZhong Medical Laboratory (Zhejiang, China) used gas chromatography (GA/T 1008.2- 2013 [1]; GB/T 29637- 2013 [2] and GB/T 29636- 2013 [3]) to verify that these samples were positive and provided relevant information, including sex, age and test results (Table S1). The test results were judged according to the Ministry of Public Security of the People's Republic of China's specification for hair sample testing of abuse-drug-related personnel.

Table S1. Information on 30 positive samples

Sample	Sex	Age	Test results		
			Morphine	Ketamine	Methamphetamine
1	F	29	+[a]	-[b]	-
2*	M	26	-	-	-
3	M	24	-	+	
4	F	28	+	-	-
5	F	42	-	-	+
6	F	30	+	-	-
7	F	24	+	-	-
8*	M	44	-	-	-
9	F	27	-	-	+
10	M	30	-	+	-
11	M	32	+	-	-
12	F	59	-	-	+
13*	F	31	-	-	-
14	F	37	+	-	-
15*	F	31	-	-	-
16	M	35	+	-	+
17*	F	26	-	-	-
18	F	34	+	-	-
19	F	25	-	+	-
20*	F	56	-	-	-
21	M	25	-	-	+

22	M	38	+	-	+
23*	F	30	-	-	-
24	F	48	-	-	+
25*	F	28	-	-	-
26*	M	54	-	-	-
27	M	25	+	-	-
28	M	53	-	+	-
29*	F	26	-	-	-
30	F	39	+	-	-

F: Female. M: Male. a: "+" means positive. When the detection result of Morphine(MOP), ketamine(KET) or methamphetamine(MET) was greater than 0.2 ng/mg, the sample was judged positive. b: "-" means negative. When the detection result of MOP, KET or MET was less than 0.2 ng/mg, the sample was judged negative. $*$: other drugs.

2. Optimization of parameters of single-component immunochromatography system

It was critical to select and optimize the detection system based on the effect of numerous parameters on the fluorescence intensity and sensitivity of the detection findings. The impacts of many system parameters were evaluated in this experiment, including the quantity of antibody labeling, the amount of fluorescent microsphere-antibody conjugate chromatography, the concentration of coated antigen and the time and buffer pH of the immune response.

2.1 Optimization of antibody labeling amount

The creation of amide bonds between amino groups and carboxyl groups is primarily responsible for the coupling of antibodies with fluorescent microspheres. Both the number of amino groups and carboxyl groups influence the development of fluorescent probes. We conjugated the same volume of fluorescent microsphere solution with 2.5, 5, 10, 20 and 40 ug/mL monoclonal antibody and then utilized the Hitachi F- 4500 fluorescence spectrometer equipment to detect negative samples. We selected the best antibody labeling amount according to the fluorescence value.

2.2 Optimization of EuNPs- mAb probes and coating concentration

We detected the negative sample through cross reaction, recorded the fluorescence value with a fluorescence reader after the appropriate reaction time and then selected the best working concentration according to the T/C value.

The concentration of fluorescently labeled antibodies on the conjugate pad and the concentration of antigens on the immunoassay strip were tuned in this investigation. We detected the negative sample through cross reaction, recorded the fluorescence value with a fluorescence reader after the appropriate reaction time and then selected the best working concentration according to the T/C value. The coating concentrations of MOP- BSA, MET- BSA and KET- BSA were 2.5, 5, 10, 15 and 20 ng/mL, respectively, and the EuNPs- mAb incorporation potency were 0.5, 1.0, 1.5, 2.0 and 2.5 mg/mL.

2.3 Optimization of reaction time and buffer pH

The immune response is a dynamic process. During the detection process, the immune response time has a certain influence on the detection technology. The negative control group BBS (0.01 M pH 8.0) and the positive experimental group (dilute the standard to 1 ng/mL) were tested and used a fluorescence reader to record the T value, C value and T/C value of the sample test strip at 3, 5, 10, 15, 20, 25 and 30 minutes. The total time of immunochromatography was 30 minutes, and the optimal working time was determined by combining the temporal dynamic changes in fluorescence intensity and inhibition rate. Competitive inhibition rate was defined as $(1- B/B_0) \times 100\%$.

Antigen antibody reactions necessitate the use of a suitable buffer system and must be performed in a pH-appropriate environment. The physical and chemical properties of antigens and antibodies are affected by pH levels that are too high or too low.

3. Performance evaluation of single-component immunoassay strips

3.1 Sensitivity

The MOP, KET and MET immunochromatographic strips were tested under optimized conditions using BBS (0.01 M pH 8.0) as a negative control. The sensitivity of the immunochromatographic strips was evaluated by diluting the three abused drugs with BBS solution and preparing standard solutions. MOP and KET standards were diluted to 0, 0.2, 0.4, 0.6, 0.8, 1, 2, 4, 6, 8, 10, 15 and 20 ng/mL, whereas MET standards were diluted to 0, 0.2, 0.4, 0.6, 0.8, 1, 2, 8, 10, 20, 25, 30 and 40 ng/mL, respectively. The experiment was performed three times for each concentration. Quantitative detection was achieved by recording the T line and C line fluorescence intensities and using the T/C value to efficiently minimize band-to-band variation and matrix effects. The X axis represents the LOG value of analyte concentration, and the Y axis shows a calibration curve for the inhibition rate.

3.2 Precision and accuracy

To evaluate the precision and accuracy of the test strips, 8 groups were chosen from the same batch for the negative control group and the positive experimental group (1 ng/mL) trial, while the inter-assay experiment employed test paper from 8 separate batches. We calculated the coefficient of variation the inter-assay and intra-assay according to the fluorescence intensity.

4. Parameter optimization results of single-component immunochromatographic test strips

To achieve a high sensitivity and fluorescence signal, several essential parameters were thoroughly tuned, including antibody labeling amount, EuNPs- mAb probe, concentration of three competitive antigens on the test line and immunization time and buffer solution pH value.

4.1 Optimization of EuNP Antibody probes

Fluorescent probes are very important in the whole immunochromatography system, and whether antibodies effectively bind to EuNPs will affect the detection effect. A transmission electron microscope (TEM) was used to identify EuNPs. The morphological changes in EuNPs before and after coupling with antibodies were observed to determine whether the coupling was successful. The state of EuNPs observed under the microscope is shown in Figure S1. We used a magnification of 50,000 times; it can be clearly observed in the field of vision that the boundaries around the non-antibody-coupled EuNPs were blurred, while the outer rings of the EuNPs- mAb coupled to the antibodies were a white membrane-like substance. The morphological changes in EuNPs before and after coupling with antibodies were significant, indicating that EuNPs were successfully coupled with antibodies.

Experiments were used to tune the concentration of three monoclonal antibodies and fluorescent microsphere labeling from 2.5 to 40 μg in order to obtain the optimal performance of EuNPs- FIA. The optimization of the antibody labeling amounts showed that the optimal labeling concentrations for the anti-MOP monoclonal antibody, anti- KET monoclonal antibody and anti-MET monoclonal antibody were 10, 20 and 10 μg/ml (Figure S2a, b, c).

4.2 Concentration optimization of coating concentration and EuNPs- mAb concentration

The concentrations of EuNPs- mAb and coated antigens were tuned using a cross-reaction experiment. The T/C value was calculated according to the optimal fluorescence signal, the optimal amount of MOP- BSA was 1.5 mg/mL and the optimal amount of EuNPs- MOP- mAb was 15 ng/mL; for MET, 1.0 mg/mL MET- BSA and 15 ng/mL EuNPs- MET- mAb; for KET, the optimal dosage was 1.5 mg/mL KET- BSA and 10ng/mL EuNPs- KET- mAb (Figure S2d–f).

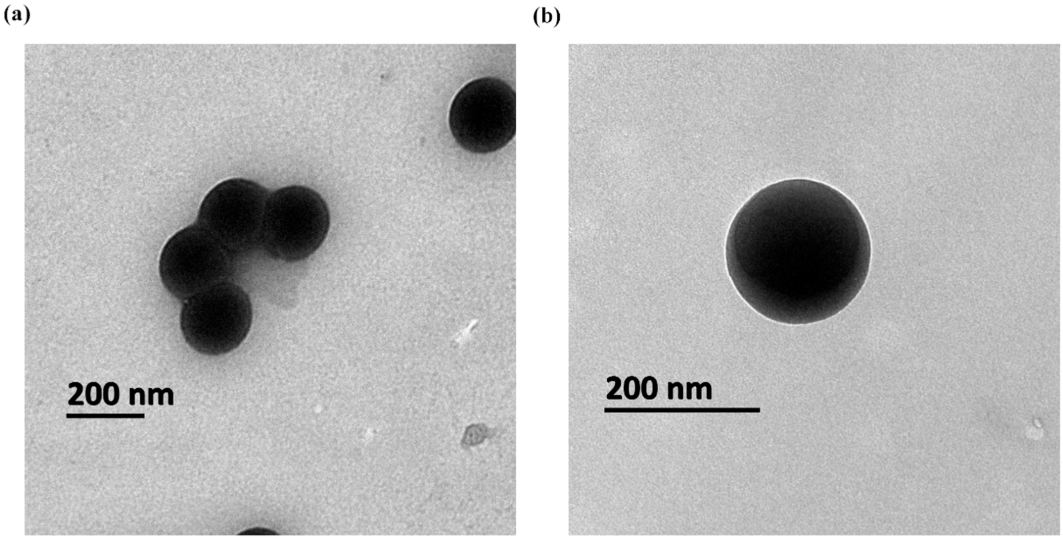

Figure S1. Identification diagram of fluorescent probe transmission electron microscope. (a): Europium nanoparticles uncoupled with antibodies. (b): Fluorescent nanoparticles after antibody coupling.

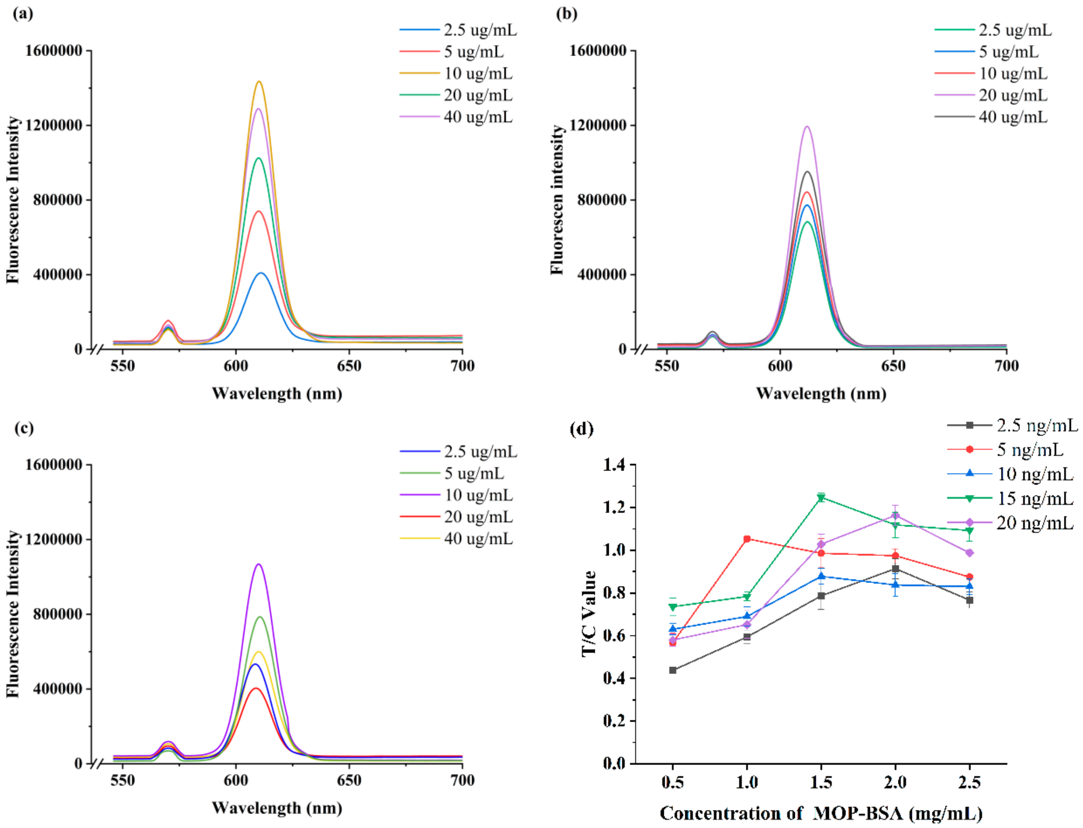

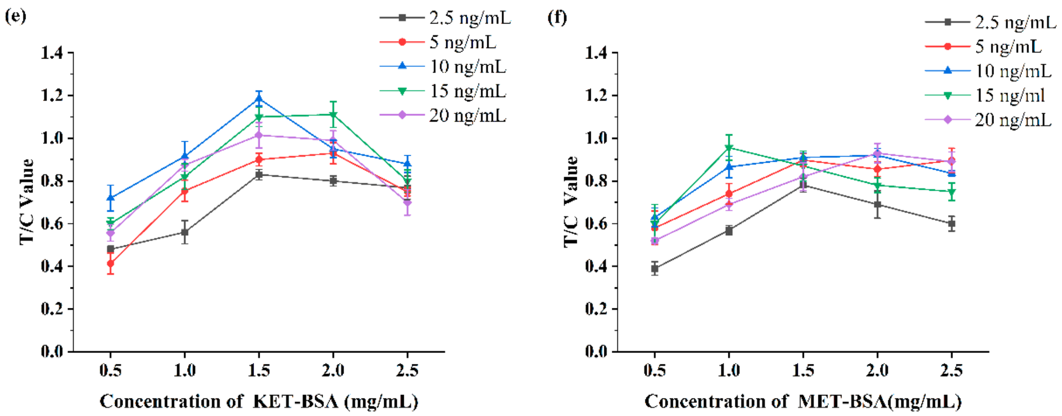

Figure S2. Optimization of EuNPs and antibody conjugation amount, EuNPs- mAb probe concentration and coating concentration. (a) The effect of MOP- mAb labeling amount on fluorescence intensity. (b) The effect of KET- mAb labeling amount on fluorescence intensity. (c) The effect of MET- mAb labeling amount on fluorescence intensity. (d) Influence of various MOP- BSA concentrations and EuNPs- mAb probe concentrations on the T/C value. (e) Influence of various KET- BSA concentrations and EuNPs- mAb probe concentrations on the T/C value. (f) Influence of various MET- BSA concentrations and EuNPs- mAb probe concentrations on the T/C value.

4.3 Optimum response time optimization and buffer pH

We used the fluorescence reader to read the fluorescence brightness of the T line and C line every few minutes until the test strip ran for 30 minutes to stop the detection. The abscissa is the immunochromatographic time, the ordinate is the ratio of T fluorescence intensity to C fluorescence intensity and the other vertical axis is the inhibition rate curve of positive samples with time. The data in Figure S3a- c demonstrate that the immune reaction time increased in 15 minutes and then stabilized 15 minutes later, and the T/C value did not rise with time. In 15 minutes, the inhibition rate, likewise, reached its peak.

The pH value can influence the antigen–antibody interactions by changing the activity of the antigen-bonding sites of the antibodies. When the pH value of the buffer solution was 8.0, the T/C value reached a peak. Therefore, the optimal pH value of the buffer solution is 8.0 (Figure S2d).

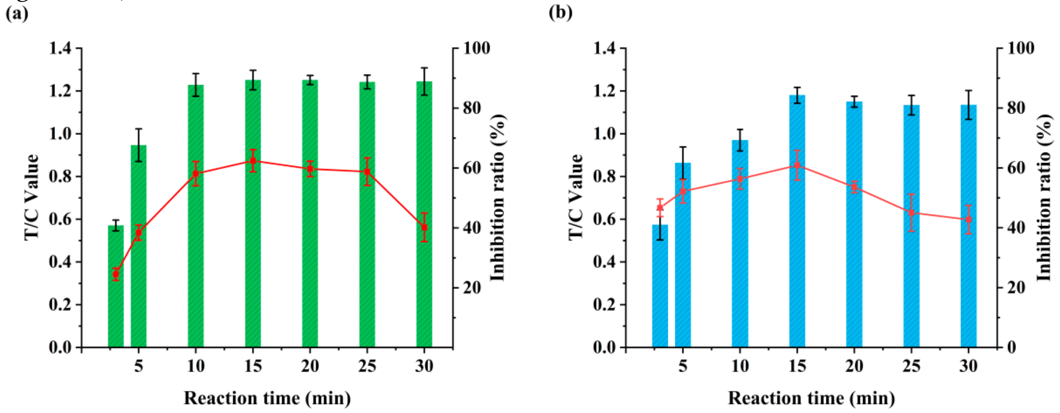

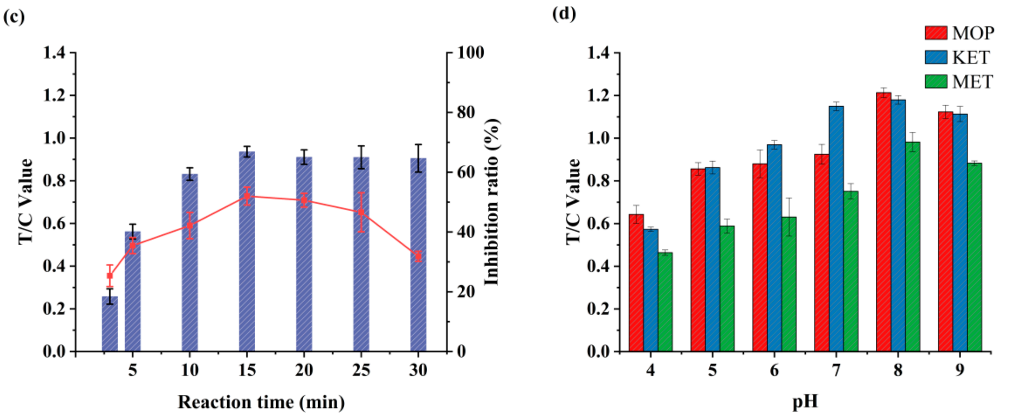

Figure S3. Optimized reaction time and buffer pH. (a) MOP reaction time. (b) KET reaction time (c) MET reaction time. (d) Buffer pH.

4.4 Performance evaluation of single-component immunochromatographic test strips

4.4.1. Sensitivity

To evaluate the detection sensitivity of a single test strip, a standard curve was constructed by reading a single test strip using the EuNPs fluorescent strip reader under optimal conditions (Figure S4a–c). The experimental results revealed that the ratio of T line to C line was negatively linked with the target analyte content in the analyte to be analyzed, which supported the competitive reaction detection concept. The ratio of T/C value at 50% inhibition concentration to blank sample T/C value is defined as IC_{50}. MOP: limit of detection (LOD)=0.116 ng/mL, IC_{50}=0.923 ng/mL and R^2=0.971 are shown in Figure S4d. KET LOD=0.068 ng/mL, IC_{50}=0.721 ng/mL and R^2=0.982 are shown in Figure S4e. Figure S4f shows MET LOD=0.113 ng/mL, IC_{50}=1.283 ng/mL and R^2=0.974. The findings demonstrate a strong relationship between the logarithm of drug misuse concentration and inhibition rate.

Hu et al. [4] established an up-converting phosphor technology-based lateral flow assay (UPT- LFA) with the sensitivities of Mop- UPT- LFA and Met- UPT- LFA at 5 and 10 ng/mL, respectively. Sun et al. [5] realized the detection of ketamine in serum by detecting Raman signals, with a detection limit of 1.64 ng/mL. At the same time, compared with other rapid detection methods shown in Table S2, the single immunochromatographic strip developed in this study had higher sensitivity to detect the three analyzed misused substances.

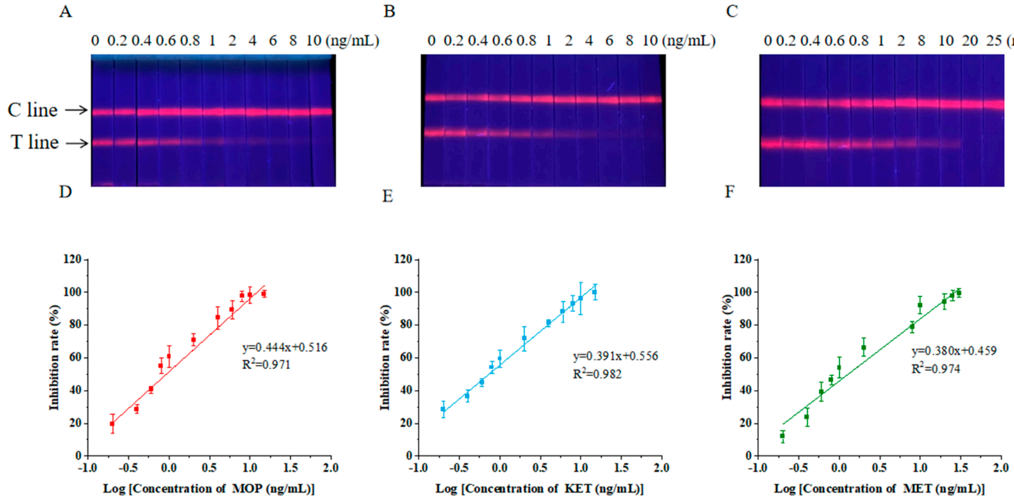

Figure S4. EuNPs- FIA single-component detection. (a) Sensitivity analysis of MOP by EuNPs- FIA. (b) Sensitivity analysis of KET by EuNPs- FIA. (c) Sensitivity analysis of MET by EuNPs- FIA. (d) The standard calibration curve for MOP by EuNPs- FIA. (e) The standard calibration curve for KET by EuNPs- FIA. (f) The standard calibration curve for MET by EuNPs- FIA.

Table S2. Comparison of the published rapid detection methods for abused drugs.

**Method**	**Test substance**	**Sample**	**LOD**	**Detection range**	**Reference**
UPT- LFA	MOP	Saliva	5 ng/mL	5- 100 ng/mL	[4]
UPT- LFA	MET	Saliva	10 ng/mL	10- 250 ng/mL	[4]
SERS	KET	Serum	1.64 ng/mL	/[a]	[5]
NFG	KET	Blood	0.1 ng/mL	0.0001- 20 μg/mL	[6]
Magnetic- LFS	COC	Urine	5 ng/mL	5–500 ng/mL	[7]
ICG	MOP	Urine	2000 ng/mL	/	[8]
QDs - LFIA	MOP	Hot Pot soup base	0.27 ng/mL	/	[9]
SERS	MET	Hair	8 ng/mL	/	[10]
LFA	THC	Oral fluid	0.01 ng/mL	/	[11]
EuNPs- FIA	MOP	Hair	0.116 ng/mL	0.116- 4.374 ng/mL	
EuNPs- FIA	KET	Hair	0.068 ng/mL	0.068- 4.224 ng/mL	This work
EuNPs- FIA	MET	Hair	0.113 ng/mL	0.113- 7.913 ng/mL	

a: Not provided
UPT- FIA: Up-converting phosphor technology-lateral flow assay
SERS: Surface-enhanced Raman scattering
NFG: Novel fluorescence gene sensor
Magnetic- LFS: Magnetic lateral flow strip
COC: Cocaine
THC: Δ9-tetrahydrocannabinol
ICG: Immunochromatography Colloidal Gold
QDs- LFIA: Quantum-dots-labeled antibody fluorescence immunoassays

4.4.2. Precision and accuracy

The reproducibility and accuracy of the single-component immunochromatographic test strips were verified by inter- and intra-assay experiments. The ratios of T line to C line in the negative control group and positive experimental group were recorded as T_0/C_0 and T/C, respectively. As shown in Table S3, the intra- and inter-assay coefficient of variation (CV) of T_0/C_0, T/C and inhibition rate of single-component MOP test strip were less than 9.36%. In Table S4, the results of the test strips used to detect KET show that the intra- and the inter-assay CV% was <9.87. Table S5 shows the results of the intra-assay and inter-assay of MET immunoassay strips, CV%<9.44.

The above experimental results prove that the single-component immunochromatographic test strips were accurate and can be used for the quantitative detection of MOP, KET and MET samples. However, in the case of the simultaneous use of multiple abused drugs, the test object was single. Therefore, the follow-up experiment improved the detection ability of the detection system by increasing the number and types of detection.

Table S3. Precision test results of single-MOP immunochromatographic test strips.

**No.**	**Intra-assay**			**No.**	**Inter-assay**		
	T_0/C_0[a]	T/C	Inhibition (%)		T_0/C_0	T/C	Inhibition (%)
1	1.18	0.54	54.23	1	1.06	0.53	50.17
2	1.22	0.60	50.86	2	1.17	0.57	50.92

3	1.13	0.59	47.84	3	1.12	0.59	47.44
4	1.27	0.61	51.89	4	1.05	0.61	42.23
5	1.22	0.57	53.23	5	1.13	0.54	51.69
6	1.12	0.60	46.52	6	1.14	0.56	50.86
7	1.14	0.61	46.66	7	0.98	0.44	55.02
8	1.09	0.54	50.30	8	1.08	0.55	48.54
CV (%)	5.33	4.94	5.85	CV (%)	5.64	9.36	7.52

a: T_0/C_0: The ratio of T line fluorescence value to C line fluorescence value of negative samples.

Table S4. Precision test results of single-KET immunochromatographic test strips.

**No.**	**Intra-assay**			**No.**	**Inter-assay**		
	T_0/C_0	T/C	Inhibition (%)		T_0/C_0	T/C	Inhibition (%)
1	1.13	0.43	61.65	1	1.11	0.54	51.63
2	1.06	0.45	57.77	2	1.06	0.42	60.19
3	1.17	0.51	56.12	3	1.17	0.48	59.02
4	1.07	0.47	56.44	4	1.08	0.45	58.67
5	1.20	0.54	55.12	5	1.19	0.47	60.78
6	1.22	0.57	53.41	6	1.10	0.51	53.79
7	1.16	0.48	58.62	7	1.09	0.49	54.97
8	1.14	0.54	52.26	8	1.26	0.53	58.23
CV (%)	4.93	9.87	5.28	CV (%)	6.42	7.42	4.51

Table S5. Precision test results of single-MET immunochromatographic test strips.

**No.**	**Intra-assay**			**No.**	**Inter-assay**		
	T_0/C_0	T/C	Inhibition (%)		T_0/C_0	T/C	Inhibition (%)
1	0.94	0.51	44.97	1	0.99	0.54	45.81
2	0.99	0.56	43.51	2	0.87	0.42	51.93
3	0.94	0.50	46.43	3	0.85	0.45	47.07
4	0.97	0.46	52.12	4	0.95	0.53	44.15
5	0.91	0.54	40.08	5	0.97	0.52	46.90
6	0.94	0.47	50.52	6	0.96	0.47	50.74
7	0.89	0.47	47.08	7	0.97	0.55	43.72
8	0.91	0.52	42.44	8	0.91	0.52	42.67
CV (%)	3.51	3.20	8.80	CV (%)	5.69	9.44	7.08

Table S6. Testing results of 530 negative hair samples.

**Detection Result (ng/mg)**	**EuNPs- FIA[a]**					
	**Number of MOP**	**Proportion of number**	**Number of KET**	**Proportion of number**	**Number of MET**	**Proportion of number**
≥0.2	0	0.00%	0	0%	0	0%
0.1- 0.2	0	0.00%	1	0.19%	3	0.57%
0.05- 0.1	3	0.57%	1	0.19%	0	0%
< 0.05	527	99.43%	528	99.62%	527	99.43%

a: repeat assay (n = 3).

Table S7. Molecule structure of abused drugs.

Drug name	Molecule structure
Morphine	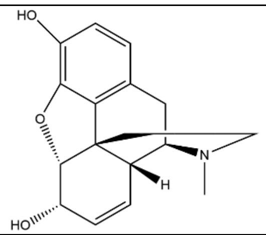
Ketamine	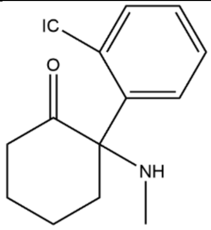
Methamphetamine	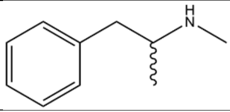
Cannabidiol	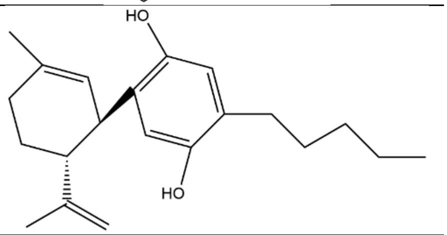
Phenobarbital	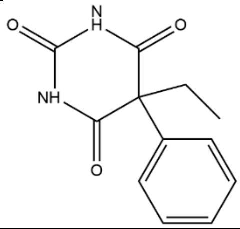
Diazepam	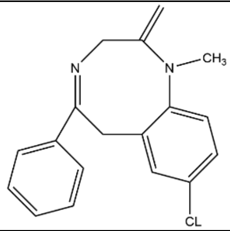
Mephedrone	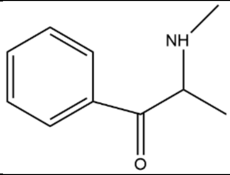
Caffeine	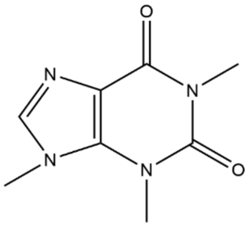
Taurine	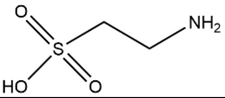

Papaverine	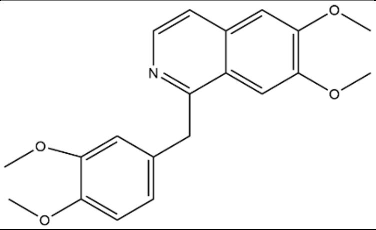
(1R,2S)-(-)-Ephedrine hydrochloride	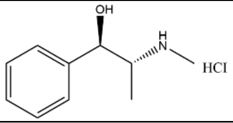
Pseudoephedrine hydrochloride	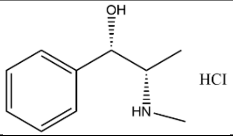
Tetrahydrocannabinol	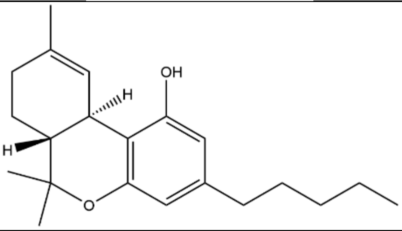
Norketamine	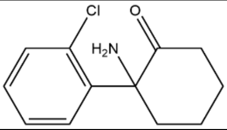
Sodium pentachlorophenol	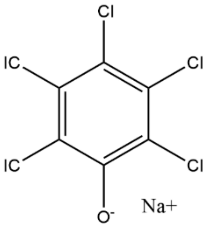
R (+)-cathinone hydrochloride	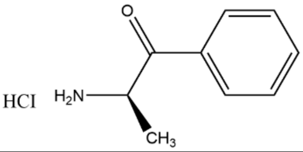
Methcathinone	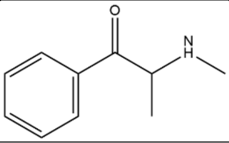
Tetrahydro-cannabinolic acid	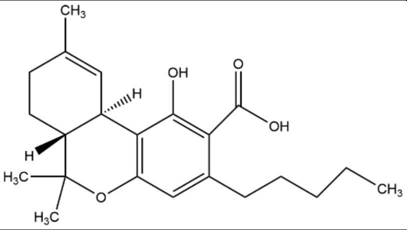
Methadone	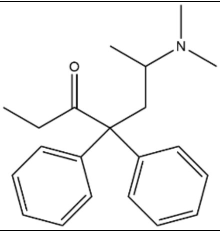

Methoxyphenamine	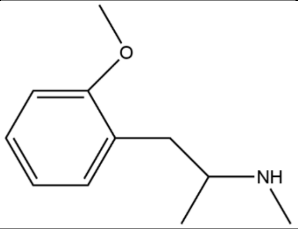
Phenylpropanolamine hydrochloride	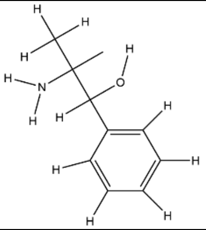
Methylenedioxymethamphetamine	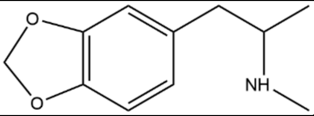
6-monoacetylmorphine	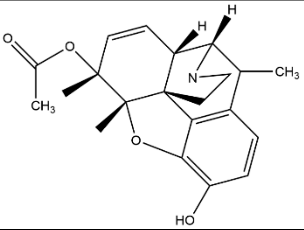
F-Ketamine	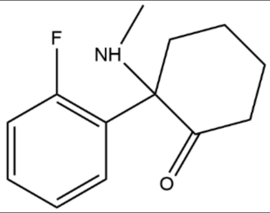

References

1. GA/T 1008.2-2013. GC and GC-MS examination methods for drugs—Part 2: Morphine Ministry of Public Security of China: Beijing, China, 2013.
2. GB/T 29637-2013. GC and GC-MS examination methods for ketamine in suspected drug. General Administration of Quality Supervision, Inspection and Quarantine of the People's Republic of China, Standardization Administration of China: Beijing, China, 2013.
3. GB/T 29636-2013. GC, HPLC and GC-MS examination methods for methylamphetamine in suspected drug. General Administration of Quality Supervision, Inspection and Quarantine of the People's Republic of China, Standardization Administration of China: Beijing, China, 2013.
4. Hu, Q.; Wei, Q.; Zhang, P.; Li, S.; Xue, L.; Yang, R.; Wang, C.; Zhou, L. An up-converting phosphor technology-based lateral flow assay for point-of-collection detection of morphine and methamphetamine in saliva. *Analyst*. 2018, 143, 4646-4654. https://dx.doi.org/10.1039/c8an00651b.
5. Sun, S.; Guan, M.; Guo, C.; Ma, L.; Zhou, H.; Wang, X.; Mi, F.; Li, J. A novel surface-enhanced Raman scattering method for simultaneous detection of ketamine and amphetamine. *RSC Adv*. 2020**,** 10, 36609-36616. https://dx.doi.org/10.1039/d0ra06839j.
6. Ding, Y.; Li, X.; Guo, Y.; Yan, J.; Ling, J.; Li, W.; Lan, L.; Chang, Y.; Cai, J.; Zha, L. Rapid and sensitive detection of ketamine in blood using novel fluorescence genosensor. *Anal Bioanal Chem*. 2017**,** 409, 7027-7034. https://dx.doi.org/10.1007/s00216-017-0650-x.
7. Wu, J.; Dong, M.; Zhang, C.; Wang, Y.; Xie, M.; Chen, Y. Magnetic lateral flow strip for the detection of cocaine in urine by naked eyes and smart phone camera. *Sensors (Basel)*. 2017**,** 17, 1286. https://dx.doi.org/10.3390/s17061286.
8. Dehghannezhad, A.; Paknejad, M.; Rasaee, M. J.; Omidfar, K. Seyyed Ebrahimi, S. S.; Ghahremani, H. Development of a nanogold-based immunochromatographic assay for

detection of morphine in urine using the Amor-HK16 monoclonal antibody. *Hybridoma (Larchmt)*. 2012**,** 31 (6), 411-6. https://dx.doi.org/10.1089/hyb.2012.0059.

9. Zhang, C.; Han, Y.; Lin, L.; Deng, N.; Chen, B.; Liu, Y. Development of quantum dots-labeled antibody fluorescence immunoassays for the detection of morphine. *J Agric Food Chem*. 2017, 65 (6), 1290-1295. https://doi.org/10.1021/acs.jafc.6b05305.
10. Peng, W.; Zong, X. Q.; Xie, T. T.; Zhou, J. W.; Yue, M. F.; Wen, B. Y.; Wang, Y. H.; Chen, J.; Zhang, Y. J.; Li, J. F. Ultrafast and field-based detection of methamphetamine in hair with Au nanocake-enhanced raman spectroscopy. *Anal Chim Acta*. 2022**,** 1235, 340531. https://dx.doi.org/10.1016/j.aca.2022.340531.
11. Plouffe, B. D.; Murthy, S. K. Fluorescence-based lateral flow assays for rapid oral fluid roadside detection of cannabis use. *Electrophoresis*. 2017, 38, 501-506. https://doi.org/10.1002/elps.201600075.
